# Covid-19 disruptions: The impact on post graduate training for periodontology

**DOI:** 10.6026/973206300210924

**Published:** 2025-05-31

**Authors:** Ena Sharma, Goyal Radhika, Krishna Sreejith, Bhardwaj Anuva, Sangha Ruhee, Sumanjit Midha, Singh Jotsaroop, Thind Simran

**Affiliations:** 1 Department of Periodontology, Rayat bahra dental college and hospital, Mohali Punjab, India; 2 Department of Oral Medicine and Radiology, Rayat Bahra Dental College and Hospital, Mohali Punjab, India; 3 Department of Periodontology, Maharishi Markandeshwar College of Dental Sciences and Research, Mullana Ambala, Haryana, India; 4 Department of Pedodontics, Rayat Bahra Dental College and Hospital, Mohali Punjab, India; 5 Department of Prosthodontics, Rayat Bahra Dental College and Hospital, Mohali Punjab, India; 6 Department of Periodontics, Rayat bahara Dental College and Hospital, Mohali Punjab, India

**Keywords:** Covid-19, periodontology, postgraduate (PG) training

## Abstract

The COVID-19 pandemic severely disrupted postgraduate (PG) teaching in periodontology, affecting academic learning, clinical training, and research productivity. While online education enhanced theoretical knowledge, restrictions on aerosol-generating procedures limited hands-on experience, especially in surgical training and implantology. A cross-sectional survey among PG students and faculty highlighted decreased clinical exposure, reduced research opportunities, and increased trainee stress. Digital tools, webinars, and virtual case discussions provided partial support but could not fully replace in-person practical learning.

## Background:

The 2019 novel coronavirus (2019-nCoV) or the severe acute respiratory syndrome corona virus 2 (SARS-CoV-2) as it is now called, is rapidly spreading from its origin in Wuhan City of Hubei Province of China to the rest of the world [1]. The World Health Organization (WHO) declared Coronavirus disease 2019 (COVID-19) as pandemic on 11th March 2020 [2]. To contain and prevent the pandemic, Government of India actively adopted various measures including national lockdown on March 25, 2020, encouraging social distancing and infection control guidelines including the use of sanitisers, masks, personal protective equipment (PPE) [3, 4]. These measures although proved better for the general population but increased the challenges for the post graduates enrolled in the dental post graduate programs. Students pursuing periodontology as their master degree are facing even more difficulties as theirs is a surgical speciality and patients are more reluctant towards surgeries even after number of cases declines. Routine out-patient department (OPD) services and elective surgeries have been either postponed or cancelled which has decreased patient footfall to hospitals. Institutes and hospitals have re-organised their services with cancellation of physical lectures, clinical exposure, journal club activities and teaching activities [5]. This has affected overall teaching and learning in an unexpected manner. The postgraduate teaching has gone through difficult times during COVID-19 pandemic. Therefore, it is of interest to discuss the problems faced by Post Graduate students due to this rapidly spreading and mutating virus.

## Material and Methods:

## Study design:

This cross-sectional study (online survey) was conducted from November 15, 2021 to February 15, 2022, to study the problems faced by postgraduate students in Periodontology in North India. A questionnaire of 50 questions was created and was circulated among students pursuing post-graduation degree in Department of Periodontology in North India including all governments and private colleges/ hospitals using online Google form available at.

## Inclusion criteria:

The target population for this study are postgraduate students who are currently pursuing Masters in Periodontology from teaching institutions within the North India.

## Data collection:

The responses submitted were checked for duplication, pooled, analysed and summarised.

## Evaluation:

The survey focussed on following points

[1] Impact on postgraduate teaching and its current status

[2] Impact on surgical training

[3] Impact on Dissertation

[4] Future implications on postgraduate training programme

[5] Mental wellbeing of postgraduate students

## Statistical analysis:

[1] Data was entered in SPSS (Statistical product and service solutions) version 26 (IBM Corp) for statistical analysis.

[2] Frequencies/percentages were summarised for all categorical variables and Chi square test, Fisher's exact test or Monte-Carlo simulation were used as appropriate.

[3] We have used Wilcoxon sign rank rest for pre (2019) and post COVID (2020) data in the same months for number of classes and cross tabulation and chi square tests for other variables for testing association.

[4] All results are described in percentage.

[5] A two-sided P value < 0.05 was considered as significant.

## Results:

## General characteristics:

The survey was sent to the most (132) Dental postgraduate students pursuing masters in department of periodontology, who could be traced in North Indian Dental teaching institutions. A total of 124 responses were collected in the survey. Total 124 responses were included in the final analysis of results, with a 93.94% response rate.

## Number of postgraduate students contacted COVID-19 (in %age):

As, third year postgraduates are involved in emergency duties compared to second year and first year postgraduates, (82%) final year postgraduates reported that they contacted COVID-19 infection. When analysed further, (32%) and (14%) for second year and first year PG's positive response was seen respectively (Figure 1 see PDF).

## Challenges faced by postgraduate students during the COVID-19 pandemic:

As reported by the respondents, (87%) postgraduates reported an issue with no physical examination of the patients, (89%) postgraduates reported issue with not able to present and learn from clinical cases, (75%) of the postgraduates mentioned that they were not able to present seminars and JC physically which disrupted the learning process. (67%) respondents claimed that they had difficulties in understanding the online mode of education which is being adapted and hence not satisfied. (72%) reported that they were not able to complete their log books which led to backlog work and hence, add on factor for stress. (45%) reported that interdepartmental meets were cancelled which affected the learning process at a broader level. As the number of cases kept on increasing, (95%) postgraduates reported fear of contacting infection. No contact and learning from the seniors was another issue reported by (87%) postgraduate students. Furthermore, (67%) students presented a problem of not having group studies and discussions affecting their learning and (5%) respondents claimed that they had no problem while adapting to the new normal way of learning amid COVID-19 pandemic (Figure 2 see PDF).

## Problems faced by postgraduate students regarding thesis:

According to the responses received, as third year postgraduates are also included, (35%) of the respondents reported that they have submitted thesis. (76%) reported difficulty in recruitment of new patients, (78%) reported issue with decrease in number of OPD which affected thesis outcome. (89%) expressed that decrease in number of surgeries affected thesis outcome whereas, (75%) expressed that decrease in follow-up affected the thesis outcome. (35%) reported that their thesis was on elective surgery, so wasn't able to continue the same, however, (24%) respondents mentioned that their thesis was changed from surgical to non-surgical owing to decline in number of cases. Also, (10%) reported no issues with the current scenario (Figure 3 see PDF).

## Problems faced in postgraduate clinical training during pandemic years:

All the Post Graduate respondents faced various problems during the covid times and some of them were reported. It included, postgraduate training has been affected due to covid pandemic (95%), decrease in surgical exposure affected learning (78%), decrease in interaction with patients affected the learning skills (67%), clinical learning has been hampered (79%), difficulty in coping up with online module of teaching as reported by (56%) respondents. Along with all these issues, other problems were also mentioned, which included the fact that physical teaching was better than online teaching (85%), postgraduates were redeployed to other places for COVID-19 sampling (56%), change in working schedule by (87%) and (5%) respondents mentioned that they didn't face any such problem (Figure 4 see PDF).

## Techniques of learning during pandemic years:

Due to suspension of offline teaching and clinics during the pandemic times, various techniques were suggested by the respondents to overcome the loss. The techniques included typhos/simulators as suggested by (62%) participants, models (42%), patient's old casts (32%) and virtual videos were suggested by (38%) (Figure 5 see PDF).

## Reasons for stress for postgraduate student in pandemic years:

As the things changed drastically, stress was observed among all the students pursuing their studies. The postgraduate respondents quoted various factors which induced stress for them. Various stress factors which could be recorded based on their responses included loss of surgical training during postgraduate as reported by (86%), loss of clinical learning during postgraduate (75%), inability to provide professional treatment to patients (45%), not being able to complete postgraduate assignments (69%), delay in completing thesis as reported by (78%) and fear of contacting COVID-19 infection was a fear factor for (84%). Apart from this, loss of confidence in performing duties were expressed by (45%), not being able to cope-up with the new normal (56%), staying away from family and friends (83%), loss of confidence in performing surgical cases (78%) and no stress was felt as reported by (3%) participants.

COVID-19 pandemic has severely affected and hampered the education protocols and training of the postgraduates enrolled in Department of Periodontology nationwide. No such study is till date available or known which analyses the problems faced by budding Periodontist in depth. Various major issues including decline footfall in clinics, suspension of aerosol producing procedures and surgeries are reported by the postgraduate students as recorded in our survey. Other problems quoted included lack of interaction with the faculty members and seniors which itself is another significant reason leading to deterioration of clinical skills. The postgraduates also have serious issues in completing their dissertations and learning from the workshops and conferences which are either postponed or cancelled. Those with surgical thesis topics are bound to change and shift to non-clinical ones due to challenges posed by pandemic. In addition, postgraduates have anxiety about not having enough confidence to set up and start with their own clinics after completing masters due to lack of clinical skills developed during postgraduate course. In nutshell, all we have analyzed is that COVID-19 pandemic has seriously affected the clinical skills, teaching skills and mental peace of postgraduates. Owing to all this, Government is requested and urged to take serious note of the plight of postgraduates and formulate possible guidelines and measures to combat the inevitable loss faced by postgraduate students (Figure 6 see PDF).

## Proposed solutions to the problems faced by postgraduates in pandemic years:

Dealing with the scenario, change in thesis topic from surgical to non-surgical was the solution given by (67%) of respondents, relaxation in sample size and duration of study was suggested by (56%), relaxation in postgraduate work from institution was another solution given by (83%). Whereas, changes in rules for postgraduate protocol from DCI was suggested by (45%), increase in virtual workshops for learning surgical skills by (87%), allotment of only non-surgical thesis by (34%), increase in virtual inter and intra departmental meets by (78%), counselling sessions for mental well-being by (67%) and adequate steps by government by (85%) were some of the solutions as suggested by the respondents (Figure 7 see PDF).

## Discussion:

The impact of COVID-19 on postgraduate (PG) training in periodontology has been profound, encompassing various aspects of education, clinical practice and research. Here are some detailed impacts:

## Challenges faced by postgraduate students during the COVID-19 pandemic:

During the COVID-19 pandemic, postgraduate students in periodontology faced numerous challenges. The closure of dental schools and clinics disrupted their clinical training, leading to limited hands-on experience. This was particularly detrimental in periodontology, where practical skills are crucial. Remote learning became the norm, but it couldn't fully replace the tactile and observational learning required in clinical practice [5]. Moreover, the pandemic led to the postponement or cancellation of elective surgeries and procedures, further limiting students' opportunities to observe and assist in complex cases. Access to patients was restricted and the fear of virus transmission made it difficult to practice routine periodontal procedures safely [6, 7]. This significantly impacted students' ability to complete required clinical hours and competencies. Research activities were also hindered due to lab closures and limited access to resources. Many students experienced delays in their research projects, affecting their academic progress and timelines for graduation. Additionally, the mental health of students suffered due to the uncertainty and stress caused by the pandemic, compounded by concerns about their future careers and job prospects in a disrupted healthcare system [8]. Overall, the pandemic severely affected the training, skill acquisition and mental well-being of postgraduate students in periodontology, posing long-term implications for their professional development.

## Problems faced by postgraduate in dissertation / thesis:

During the COVID-19 pandemic, postgraduate students in periodontology faced numerous challenges in conducting their dissertations. Firstly, access to clinical settings was severely restricted due to lockdowns and social distancing measures, limiting opportunities for hands-on research and patient interactions essential for clinical studies. Many dental clinics and hospitals either closed or operated at reduced capacities, affecting the availability of case studies and patient follow-ups [9]. Secondly, the pandemic disrupted the supply chain of essential dental materials and equipment, causing delays and shortages that hindered experimental work. This impacted the timeline and scope of research projects, often necessitating changes in study design or postponement [10, 11]. Thirdly, travel restrictions and quarantine protocols impeded fieldwork and data collection, particularly for studies requiring multi-center collaboration or external validation. The shift to virtual learning and remote supervision posed additional difficulties. Many students struggled with limited access to institutional resources such as libraries, laboratories and study spaces. Moreover, the psychological stress and anxiety related to the pandemic affected students' productivity and mental health, complicating their ability to focus on rigorous academic work. These factors collectively led to significant delays, increased stress and compromised the quality of dissertations in periodontology during the COVID-19 crisis [12, 13- 14].

## Impact on mental well-being:

The COVID-19 pandemic has profoundly affected the mental well-being of postgraduate students in periodontology. Increased stress and anxiety stemmed from fears about the virus, concerns for personal and family health and the pressure to adapt to online learning and virtual clinics [15]. The disruption of clinical training, with the suspension of non-emergency dental procedures, raised worries about skill acquisition and meeting graduation requirements, impacting students' confidence and competence. Isolation due to social distancing and lockdowns exacerbated feelings of loneliness and depression, as students lost in-person interactions with peers and mentors. Uncertainty regarding future prospects, including delays in exams, thesis submissions, job opportunities and heightened anxiety. Financial strain, from reduced part-time income or family financial stress, further contributed to their mental burden [16]. To scope, students adopted various strategies, such as engaging in physical activities, developing new hobbies and seeking virtual support groups. Educational institutions responded by offering virtual counselling services, online resources and platforms for peer interaction. Despite these efforts, the pandemic has underscored the need for robust mental health support systems for postgraduate students in periodontology [17].

## Future implications in Covid-19 postgraduate training protocol:

The COVID-19 pandemic has reshaped postgraduate (PG) training programs in periodontology, leading to several significant future implications. One primary change is the increased use of technology, with virtual learning platforms becoming essential. Postgraduate programs will continue to integrate online classrooms, webinars and virtual assessments. Tele-dentistry will gain prominence, requiring students to become proficient in remote consultation technologies [18-19] Enhanced infection control training will be crucial. Postgraduate programs will incorporate stringent protocols for PPE usage, sterilization and disease management. Curriculums will expand to include comprehensive modules on infection control, epidemiology and public health. Hybrid learning models, combining online theoretical education with in-person practical sessions, will become the norm. This approach ensures hands-on experience while maintaining social distancing. Simulation-based training using dental simulators and virtual reality will also rise, reducing reliance on patient-based training in early learning stages [20]. The research focus within periodontology will shift, with increased attention on COVID-19's oral manifestations, its impact on periodontal health and periodontists' roles in managing these conditions. Public health research will gain importance, emphasizing periodontists' contributions to community health during pandemics. Mental health and well-being will be prioritized, with programs implementing support systems for stress and burnout. Resilience training will be integrated to help students cope with future challenges [18]. Flexible training structures will emerge, offering adaptable schedules to accommodate disruptions and potentially extending program durations to ensure comprehensive training. Interdisciplinary collaboration will increase, fostering cross-disciplinary learning and research opportunities that explore the broader implications of infectious diseases on oral health. Regulatory and accreditation changes will follow, with updated standards requiring pandemic preparedness and response training. Continuous assessment methods will ensure competency despite potential disruptions. These adaptations will make postgraduate training programs in periodontology more resilient, flexible and comprehensive, equipping graduates to face diverse challenges effectively [21]. The COVID-19 pandemic has driven long-term changes and innovations in postgraduate training in periodontology. Programs now emphasize virtual learning, hybrid models combining online and in-person sessions and enhanced infection control protocols. Tele-dentistry and simulation-based training using VR and dental simulators are becoming standard. Curriculums include more on public health, epidemiology and mental health support. Flexible schedules and interdisciplinary collaborations are encouraged. Regulatory bodies are updating standards for pandemic preparedness. These changes ensure that future periodontists are well-equipped with the skills and resilience needed to manage both routine and crisis situations effectively.

## Conclusion:

The COVID-19 pandemic has severely impacted postgraduate training in periodontology, leading to reduced clinical exposure, suspension of aerosol-generating procedures and limited faculty interactions, all of which have hindered skill development. Challenges in completing dissertations, attending workshops and adapting surgical thesis topics have further affected postgraduate education, raising concerns about future professional competence. Hence, policies should be established at both institutional and government levels to ensure resilient educational frameworks, incorporating blended learning, structured clinical training and contingency plans for academic continuity during pandemics.
